# MRI radiomics and potential applications to glioblastoma

**DOI:** 10.3389/fonc.2023.1134109

**Published:** 2023-02-17

**Authors:** Grayson W. Hooper, Daniel T. Ginat

**Affiliations:** ^1^ Landstuhl Regional Medical Center, Department of Radiology, Landstuhl, Germany; ^2^ University of Chicago, Department of Radiology, Chicago, IL, United States

**Keywords:** MRI, glioblastoma, radiomic analysis, radiomics, radiology, gliomas

## Abstract

MRI plays an important role in the evaluation of glioblastoma, both at initial diagnosis and follow up after treatment. Quantitative analysis *via* radiomics can augment the interpretation of MRI in terms of providing insights regarding the differential diagnosis, genotype, treatment response, and prognosis. The various MRI radiomic features of glioblastoma are reviewed in this article.

## 1 Introduction

MRI is routinely used to formulate a differential diagnosis at the initial presentation of patients with glioblastoma, help guide treatment planning, and follow up treatment response. However, the interpretation of MRI findings in these situations can be challenging. With respect to oncologic imaging, radiomics can provide useful insights regarding tumor characteristics. Specifically, radiomics involves extracting quantitative information from the images, such as texture features, often using artificial intelligence. Reproducible radiomic features of particular tumor types can serve as imaging biomarkers. In this article, various types of radiomic analysis pertaining to glioblastoma depicted on MRI are reviewed.

Glioblastoma is a heterogeneous neoplasm both in imaging appearance and internal genotype. Tissue biopsy from one part of the tumor may not reflect histology in another (1). While this may not have much practical implication beyond explaining treatment failure now, it certainly will in the future. The heterogeneity of glioblastoma is a topic of significant research, as identification of its variable internal constituents will be vital in development of effective therapeutics. This article reviews the role that quantitative MRI may have through the use of radiomics for characterizing glioblastoma heterogeneity and differentiating glioblastoma from other entities.

## 2 Diagnosis and differential considerations

The appearance of glioblastoma on MRI can sometimes be difficult to distinguish from other neoplasms and even non-neoplastic conditions, such as lower-grade gliomas, primary CNS lymphoma, tumefactive multiple sclerosis (MS), and various infections. Even using advanced MRI techniques such as arterial spin labeling, magnetic resonance spectroscopy (MRS), and perfusion imaging, tissue sampling is typically performed for definitive diagnosis.

Through the computational analysis of MRI at the pixel level, radiomics may help establish a certain diagnosis. Radiomics uses statistical methods to extract useful information from medical images that is otherwise generally beyond human perception. This information, called *features*, comes in two varieties: semantic and agnostic. Semantic features are the bread and butter of the radiologist. They are common descriptors of radiologic findings such as size and morphology, which guide management, and which have been correlated to gene expression (2, 3). Agnostic features are aptly named—they are esoteric mathematical derivations that reflect patterns beyond human perception. As previously mentioned, features are prone to variation, highlighting the need for standardization in order to identify stable ones (4). However, many features are also useless, being redundant or irrelevant, which contribute only noise to a model. Through a series of filtration methods, which are well-described by Papanikolaou and colleagues, features can be curated to form a radiomics model (5). However, the model must still be externally validated and statistically correlated to a clinical endpoint. It must be reproducible and answer a clinical question. Only then can it be a useful signature.

Most radiomics studies are retrospective, which is useful for building and testing models; however, they are limited in their clinical application. Regardless, there have been promising advances in delineating glioblastomas from other disease processes. For example, Conte and colleagues showed that 100 primary semantic and agnostic features based on T2-weighted images were able to delineate glioblastoma from tumefactive MS with a 0.83 accuracy and 0.86 area under the ROC curve for the best set of hyperparameters (6). Another retrospective study utilized multiparametric MRI sequences, including T1, T2, and postcontrast imaging in order to delineate glioblastoma from primary CNS lymphoma. In this study, regions encompassing the solid tumor component as well as the tumor and the peritumoral edema were selected. Of the 127 total features available, just 15 were selected, which still rendered an area under the curve of 0.979, a sensitivity of 0.938, and a specificity of 0.944 (7).

A study by Kunimatsu et al. compared the T1 postcontrast textural features of glioblastoma to primary CNS lymphoma. Their principal component analysis revealed that only four of these features—entropy, median, run length non-uniformity, and run percentage—were the predominant features of the dataset, which could explain the variance of the feature set (8). In doing so, this study suggested specific features for future analysis that may be helpful in delineating the two disease processes and address the problem of overfitting.

Another potential scenario with important clinical management implications is differentiating glioblastoma versus a solitary intracranial metastasis, which can also demonstrate edema, heterogeneity, and ring-enhancing features identical to glioblastoma. It is here too that radiomics has shown significant promise. Many of these models utilize similar textural analysis algorithms to accomplish the task (9–11). Indeed, a 2022 meta-analysis involving seventeen studies encompassing 1,717 total patients has shown the pooled sensitivity and specificity of radiomics in differentiating glioblastoma from metastasis are both 84 percent (12). In addition to MRI, more recent studies have shown that multimodality, integrative models have improved diagnostic accuracy. Zhang and colleagues showed that an integrated model including contrast-enhanced T1, T2-weighted images, diffusion-weighted images, ADC maps, and 18F-FDG PET obtained areas under the curve of 0.98 and 0.93, sensitivities of 0.925 and 0.835, and specificities of 0.987 and 0.849 for the training and validation sets (13).

Given that MRI has excellent tissue contrast and can provide diverse information about water diffusivity, blood flow, the state of the blood brain barrier, and cellularity, it follows that radiomic analysis of these data could provide further valuable information. Multiple studies have shown promise in differentiating low-grade gliomas from high-grade ones using textural analysis (14–17). Others have shown that radiomic phenotyping of glioblastoma improved survival prediction (18, 19). Yet others have demonstrated potential use in predicting treatment outcomes and assessing pseudoprogression in high-grade gliomas (20, 21). These are remarkable results considering that the analyzed features are mostly beyond human perception.

## 3 Treatment planning

Surgery, temozolomide, and radiotherapy are the current standard of care for treating patients with glioblastoma. Unfortunately, the mean survival time of these patients remains about 15 months. There are multiple ideas as to why glioblastoma is so lethal and resilient. One contributing factor may be the cancer stem cell, which facilitates a mesenchymal transformation that allows the tumor to better infiltrate the brain. These cancer stem cells may also adapt to temozolomide therapy directly. The second idea is the tumor microenvironment. Glioblastoma is comprised of approximately 40% non-malignant cells, which act as a support network. These cells are immunosuppressive and generate a vascular-proliferative environment to help the tumor grow. Another consideration is the heterogeneity of the tumor in general. The phenotypes and genotypes of glioblastomas vary throughout their architecture and change as the disease progresses and as treatments are applied. Radiomics may play an important role here in characterizing the tumor both phenotypically and ultimately genotypically (radiogenomics), which would permit a tailored and dynamic therapeutic response. However, first biomarkers must be identified.

There are several critical mutations and molecular alterations in glioblastomas, which may be actionable biomarkers of the disease and are the subjects of intense research. The first of these is mutation of the epidermal growth factor receptor (EGFR), which is seen in about 60% of primary glioblastomas and results in a more aggressive tumor phenotype (2). In a 2017 study, Zinn and colleagues quantified the mutually exclusive and significant (false discovery rate 0.05) MRI texture features of EGFR, TP53, and PTEN mutations (22). They also analyzed the upregulated genes that were unique to each mutation versus their wildtype comparisons. The biofunctions of the mutated gene clusters were linked to predominant functions, showing TP-53-angiogenesis, PTEN-invasion, and EGFR-immune response. Consensus clustering demonstrated similar correlation matrices of the radiomic features of mutants versus wildtype mutations and the corresponding gene expressions. These findings suggesting a link between radiomic texture features and biologic function and could prove important in treatment planning, though specific compounds targeting EGFR have not yet proven effective. Early tyrosine kinase inhibitors targeting EGFR have shown little clinical efficacy in glioblastoma patients, though research is ongoing, including preclinical trials in third generation EGFR irreversible inhibitors (23, 24).

Another mutation significant to the pathogenesis of gliomas, including glioblastoma, is isocitrate dehydrogenase-1 (IDH-1) on chromosome 2. Glioblastoma can arise *de novo* or develop from lower grade gliomas through sequential mutations (25). Wildtype glioblastoma is far more common than mutational, and is considered the more aggressive form, which responds poorly to current therapies and has a dismal five-year prognosis. Glioblastomas with IDH-1 mutations account for 73% of tumors and are reported to have a slightly better prognosis due to their enhanced response to radiotherapy and temozolomide (26). Regardless, IDH-1 mutants and wildtype tumors have different metabolic profiles, suggesting that each type may have unique therapeutic vulnerabilities (27). One such potential vulnerability is seen in the IDH1^R132H^ mutational subtype, which has significantly improved prognosis versus other gliomas, and overexpresses microRNA mIR-128. This microRNA suppresses glioma cell proliferation *in vitro*, possibly accounting for the survival benefit *in vivo*, and making it a potential therapeutic target (28). Therefore, imaging differentiation of this mutation remains a valuable goal. Multiple studies have had varying degrees of success in the use of radiomics for identifying IDH mutations (29–31). Of further note, IDH-1 mutation also has other diagnostic implications, as IDH-1 mutants produce 2-Hydroxyglutarate, which can be detected on MRS (32).

O6-methylguanine–DNA methyltransferase (MGMT) is a DNA-repair enzyme that repairs damage caused by alkylating agents. Production of this enzyme is inhibited by methylation of its promoter region; therefore, methylation status can predict a patient’s response to temozolomide therapy (33). Indeed, MGMT methylation status is the dominant predictor of increased survival in patients with glioblastoma, even showing significant value in patients with unresectable disease (34). Consequently, utilizing MRI to assess this gene’s methylation status would have prognostic and treatment value. Sasaki and colleagues used T1 precontrast, T1 postcontrast, and T2-weighted images to analyze the radiomics features of 201 preoperative patients with glioblastoma. Despite being able to stratify low and high-risk patients based on 22 features correlated with prognosis, this study did not display sufficient accuracy for the prediction of MGMT methylation status (35). A similar study by Chen et al. (though focused on diffuse gliomas) was able to predict methylation status of the tumors utilizing deep learning and features extracted from postcontrast T1 and ADC imaging (36).

## 4 Post-therapeutic assessment

Complete surgical excision of glioblastomas is extremely rare and they almost invariably recur, often in the surgical bed. Therefore, routine monitoring of patients is essential. Unfortunately, it can be difficult to differentiate true disease progression from radiation-associated pseudo-progression on routine MR imaging and its negative predictive value is not sufficient for clinical use (37). This is a significant problem as pseudo-progression occurs in up to a third of patients according to a 2017 meta-analysis (38). Advanced MR techniques, to include perfusion-weighted imaging and MRS have proven useful in supplementing routine sequences with a combined accuracy of about 90 percent (39). Indeed, a recent meta-analysis of 30 studies showed that dynamic susceptibility contrast (DSC) MRI and DWI had the best sensitivity and specificity in differentiating true progression from pseudoprogression in high-grade gliomas (40). Radiomics again may prove useful in this setting. Two studies have investigated algorithmic assessment of routine MR images (41, 42). These showed diagnostic accuracies of around 70%, performing better than the neuroradiologists. Similarly, another study that integrated Ktrans and relative cerebral blood volume metrics from PWI sequences attained an accuracy of about 91% (43).

## 5 Limitations of radiomics

There are unique challenges in the application of radiomics to MRI. Unlike CT where attenuation in Hounsfield Units is directly correlated to tissue density, the signal obtained from MRI is unitless and can variably reflect tissue properties. MRI field strength, pulse sequences, as well as manufacturer differences have been shown to affect radiomics features (44, 45). Motion artifact on MRI can also be significant as the sequences take longer to obtain. Additionally, magnetic field inhomogeneity from intrinsic and extrinsic sources can affect image acquisition. Consequently, there is a greater potential for imaging variability in MRI than CT, which could affect the robustness of imaging features. Various computational postprocessing techniques can be used to normalize MRI imaging data. These techniques have been shown to improve the prognostic value and stability of radiomics features in the setting of glioblastoma, though only 8% (113/1404) remained stable in a multicenter study (46).

Radiomics is also prone to multiple forms of error intrinsic to high level statistical analysis. Specifically, *feature-based* radiomics requires the selection and analysis of a region-of-interest. This is not only prone to human variability if it is not assisted by artificial intelligence, but it may also result in *overfitting*, which occurs if excessive high-dimensional data is applied to a small cohort. The result is a model that is “self-assessing” in that it corresponds to a single set of data but is otherwise useless in predicting outcomes elsewhere. Indeed, a 2022 study by Gidwani and colleagues revealed two marked shortcomings in contemporary radiomics literature: improper data partitioning and unproductive feature association. The first resulted in data leakage during the training, validation, and/or testing phases of these studies. The second resulted in feature self-association and overfitting. When these errors were applied to randomly generated features based on real-world data sets, they discovered a spurious combined 1.4 factor performance boost to radiomics models, indicating random chance after correction (47). Consequently, future radiomics studies must be large and multicenter with careful attention to features selection *and* data partitioning in order to mitigate this issue and create reproducible models (48). In deep learning radiomics, also known as deep radiomics, convoluted neural networks (CNNs) or autoencoders search for relevant patterns/features in a dataset without image segmentation. Therefore, the dataset must be large to prevent overfitting, though transfer learning in which a neural network is trained on an outside dataset, can be used to mitigate this shortcoming (49). Unfortunately, deep learning also has the “black box” shortcoming in which the AI presents information that is uninterpretable, which cannot be linked to findings of clinical relevance.

As mentioned, radiomics thrives or fails based on the reproducibility of its features. Certainly, when the success of the analysis is incumbent upon data reviewed at the pixel level, it is essential that imaging acquisition, segmentation, and analysis techniques are standardized. Unfortunately, most studies to his point have been small and retrospective, which have been valuable in early proof-of-concept of the field but insufficient for clinical validation. As a result, more recent attempts at reference value standardization have been undertaken. The Image Biomarker Standardization Initiative (IBSI) is one such example in which multiple teams established reference values for 169 radiomics features utilizing a phantom and publicly available lung cancer imaging. These values were then validated on multimodality imaging of soft tissue sarcomas with 167 of the features demonstrating good or excellent reproducibility (50).

Certainly, radiomics does have significant challenges. But such an emerging technology could prove invaluable to the field of neurooncology. The concept of a virtual biopsy is alluring, especially with the heterogeneity of glioblastoma. Indeed, as contemporary molecular genetics works to elucidate gene expression in glioblastomas and reveal potential sites for targeted therapy, it is essential that medical imaging synchronously works to reveal these biomarkers. Radiogenomics in this case would accomplish two tasks. The first, of course, to better characterize the glioblastoma. The second would be to link the radiomics features to a biologic basis. Liu and colleagues accomplished this in patients with low-grade gliomas, showing that T2-weighted radiomics features of progression-free survival were significantly associated with apoptosis, immune response, cell proliferation, and vascular development (51). A 2020 paper by Franco and colleagues outlined a future study that aims to use machine learning to analyze magnetic resonance spectroscopy (MRS) metabolite profiles in order to predict tumor types (52). Thus, by linking the abstract semantic features of radiomics to a tangible biologic basis, such endeavors could in turn improve understanding and acceptance of the technology. Nonetheless, radiomics remains a largely experimental field that has not been routinely adopted for clinical use. This is due to difficulty reproducing results and a lack of understanding between the radiomics signatures and the underlying biology, which undermines trust (53).

## 6 Future direction and conclusion

Radiomics is an evolving field. It is currently in its discovery phase with a preponderance of retrospective studies serving as proof-of-concept. As it stands now, radiomics has extraordinary potential to revolutionize diagnostic medicine. From the macro level of differentiating tumor types and various non-oncogenic pathologies to the micro level of tumor histology down to possibly radiogenomics, the use of computers to supplement the human eye will grow.

Ultimately, the computational analysis of MRI *via* radiomics and artificial intelligence is promising for characterizing glioblastoma, particularly using multimodality combinatorial models. However, radiomics is limited by the lack of standardization, which hinders reproducibility. Therefore, it is imperative that routine techniques in both imaging acquisition and segmentation are established. Radiomics also must explain the biologic underpinnings of its features in order to gain trust and move towards clinical translation (54). Studies such as that of Zinn and colleagues hint at such explanations and add unique value to the credibility of this growing field. Nevertheless, more prospective examinations of radiologic imaging correlation to controlled genetic gain of function are warranted.

## Author contributions

The authors are both responsible for drafting and editing the article. All authors contributed to the article and approved the submitted version.

## References

[1] Parker N Hudson A Khong P Parkinson JF Dwight T Ikin RJ. Intratumoral heterogeneity identified at the epigenetic, genetic and transcriptional level in glioblastoma. Sci Rep (2016) 6(22477). doi: 10.1038/srep22477 PMC4778014 26940435

[2] Diehn M Nardini C Wang DS Kuo MD McGovern S Liang Y. Identification of noninvasive imaging surrogates for brain tumor gene-expression modules. PNAS (2008) 105(13):5213–8. doi: 10.1073/pnas.0801279105 PMC2278224 18362333

[3] Hasse A Dapash M Jeong Y Ansari SA Carroll TJ Lesniak M. Correlation of post-contrast T1-weighted MRI surface regularity, tumor bulk, and necrotic volume with Ki67 and p53 in glioblastomas. Neuroradiology (2019) 61:861–7. doi: 10.1007/s00234-019-02204-1 PMC6693669 31020343

[4] Zwanenburg A Leger S Agolli L Pilz K Troost EGC Richter C. Assessing robustness of radiomic features by image perturbation. Sci Rep (2019) 9(1):614. doi: 10.1038/s41598-018-36938-4 30679599 PMC6345842

[5] Papanikolaou N Matos C Koh DM. How to develop a meaningful radiomic signature for clinical use in oncologic patients. Cancer Imag (2020) 20(1):33. doi: 10.1186/s40644-020-00311-4 PMC7195800 32357923

[6] Conte GM Eckel-Passow JE Tobin WO Decker P Lachance DH Jenkins RB. (2021). Differentiation of tumefactive multiple sclerosis and glioblastoma using radiomics features extracted from magnetic resonance imaging and machine learning [abstract], in: Proceedings of the AACR Virtual Special Conference on Artificial Intelligence, Diagnosis, and Imaging, Philadelphia (PA): AACR, 2021 Jan 13-14, Vol. 27.

[7] Kim Y Cho HH Kim ST Park H Nam D Kong DS. Radiomics features to distinguish glioblastoma from primary central nervous system lymphoma on multi-parametric MRI. Neuroradiology (2018) 60(12):1297–305. doi: 10.1007/s00234-018-2091-4 30232517

[8] Kunimatsu A Kunimatsu N Kamiya K Watadani T Mori H Abe O. Comparison between glioblastoma and primary central nervous system lymphoma using MR image-based texture analysis. Magn Reson Med Sci (2018) 17(1):50–7. doi: 10.2463/mrms.mp.2017-0044 PMC5760233 28638001

[9] Artzi M Bressler I Bashat DB. Differentiation between glioblastoma, brain metastasis and subtypes using radiomic analysis. J MRI. (2019) 50(2):519.528. doi: 10.1002/jmri.26643 30635952

[10] Chen C Ou X Wang J Guo W Ma X. Radiomics-based machine learning in differentiation between glioblastoma and metastatic brain tumors. Front Oncol Sec. Cancer Imaging Image-direct Interventions (2019) 9):806. doi: 10.3389/fonc.2019.00806 PMC6714109 31508366

[11] Su CQ Chen XT Duan SF Zhang JX You YP Lu SS. A radiomics-based model to differentiate glioblastoma from solitary brain metastases. Clin Radiol (2021) 76(8):629.e11–629.e18. doi: 10.1016/j.crad.2021.04.012 34092362

[12] Li Y Liu Y Liang Y Wei R Zhang W Wang Y. Radiomics can differentiate high-grade glioma from brain metastasis: a systematic review and meta-analysis. Eur Radiol (2022) 32(11):8039–51. doi: 10.1007/s00330-022-08828-x 35587827

[13] Zhang L Yao R Gao J Tan D Yang X Wen M. An integrated radiomics model incorporating diffusion-weighted imaging and ^18^F-FDG PET imaging improves the performance of differentiating glioblastoma from solitary brain metastases. Front Oncol.Sec. Neuro-Oncol Neurosurg Oncol (2021) 11):732704. doi: 10.3389/fonc.2021.732704 PMC8435895 34527594

[14] Kobayashi K Miyake M Takahashi M Hamamoto R. Observing deep radiomics for the classification of glioma grades. Sci Rep (2021) 11(1):10942. doi: 10.1038/s41598-021-90555-2 34035410 PMC8149679

[15] Tian Q Yan LF Zhang X Zhang X Hu YC Yu H. Radiomics strategy for glioma grading using texture features from multiparametric MRI. J Magn Reson Imag (2018) 48(6):1518–28. doi: 10.1002/jmri.26010 29573085

[16] Xie T Chen X Fang J Kang H Xue W Tong H. Textural features of dynamic contrast-enhanced MRI derived model-free and model-based parameter maps in glioma grading. J Magn Reson Imag (2018) 47(4):1099–111. doi: 10.1002/jmri.25835 28845594

[17] Malik N Geraghty B Dasgupta A Maralani PJ Sandhu M Detsky J. MRI Radiomics to differentiate between low grade glioma and glioblastoma peritumoral region. J Neurooncol. (2021) 155(2):181–91. doi: 10.1007/s11060-021-03866-9 34694564

[18] Bae S Choi YS Ahn SS Chang JH Kang S. Radiomic MRI phenotyping of glioblastoma: Improving survival prediction. Radiology (2018) 289(3):797–806. doi: 10.1148/radiol.2018180200 30277442

[19] Yan J Zhang B Zhang S Cheng J Liu X Wang W. Quantitative MRI-based radiomics for noninvasively predicting molecular subtypes and survival in glioma patients. NPJ Precis Oncol (2021) 5(1):72. doi: 10.1038/s41698-021-00205-z 34312469 PMC8313682

[20] Kickingereder P Götz M Muschelli J Wick A Neuberger U Shinohara RT. Large-Scale radiomic profiling of recurrent glioblastoma identifies an imaging predictor for stratifying anti-angiogenic treatment response. Clin Cancer Res (2016) 22(23):5765–71. doi: 10.1158/1078-0432.CCR-16-0702 PMC5503450 27803067

[21] Mammadov O Akkurt BH Musigmann M Ari AP Bloemer DA Kasap DNG. Radiomics for pseudoprogression prediction in high grade gliomas: added value of MR contrast agent. Heliyon (2022) 8(8):e10023. doi: 10.1016/j.heliyon.2022.e10023 35965975 PMC9364026

[22] Zinn P Singh S Kotrotsou A Abrol S Thomas G Mosley J. Distinct radiomic phenotypes define glioblastoma TP53-PTEN-EGFR mutational landscape. Neurosurgery (2017) 64(CN_suppl_1):203–10. doi: 10.1093/neuros/nyx316 PMC6433436 28899058

[23] Westphal M Maire CL Lamszus K. EGFR as a target for glioblastoma treatment: An unfulfilled promise. CNS Drugs (2017) 31(9):723–35. doi: 10.1007/s40263-017-0456-6 PMC5573763 28791656

[24] Reardon DA Nabors LB Mason WP Perry JR Shapiro W Kavan P. BI 1200 36 trial group and the Canadian brain tumour consortium. phase i/randomized phase II study of afatinib, an irreversible ErbB family blocker, with or without protracted temozolomide in adults with recurrent glioblastoma. Neuro Oncol (2015) 17(3):430–9. doi: 10.1093/neuonc/nou160 PMC4483093 25140039

[25] Urbańska K Sokołowska J Szmidt M Sysa P. Glioblastoma multiforme - an overview. Contemp Oncol (Pozn). (2014) 18(5):307–12. doi: 10.5114/wo.2014.40559 PMC4248049 25477751

[26] Han S Liu Y Cai SJ Qian M Ding J Larion M. IDH mutation in glioma: molecular mechanisms and potential therapeutic targets. Br J Cancer. (2020) 122(11):1580–9. doi: 10.1038/s41416-020-0814-x PMC7250901 32291392

[27] Garrett M Fujii Y Osaka N Sasaki A. Emerging roles of wild-type and mutant IDH1 in growth, metabolism and therapeutics of glioma. In: Debinski W, editor. Gliomas [Internet]. Brisbane (AU Exon Publications (2021). doi: 10.36255/exonpublications.gliomas.2021.chapter4 34038049

[28] Nie QM Lin YY Yang X Shen L Que SL. IDH1R¹³²H decreases the proliferation of U87 glioma cells through upregulation of microRNA-128a. Mol Med Rep (2015) 12(5):6695–701. doi: 10.3892/mmr.2015.4241 PMC4626131 26324126

[29] Macyszyn L Akbari H Pisapia JM Da X Attiah M Pigrish V. Imaging patterns predict patient survival and molecular subtype in glioblastoma *via* machine learning techniques. Neuro Oncol (2016) 18(3):417–25. doi: 10.1093/neuonc/nov127 PMC4767233 26188015

[30] Wu S Meng J Yu Q Li P Fu S. Radiomics-based machine learning methods for isocitrate dehydrogenase genotype prediction of diffuse gliomas. J Cancer Res Clin Oncol (2019) 145(3):543–50. doi: 10.1007/s00432-018-2787-1 PMC6394679 30719536

[31] Calabrese E Villanueva-Meyer JE Cha S. A fully automated artificial intelligence method for non-invasive, imaging-based identification of genetic alterations in glioblastomas. Sci Rep (2020) 10(11852). doi: 10.1038/s41598-020-68857-8 PMC7366666 32678261

[32] Andronesi OC Kim GS Gerstner E Batchelor T Tzika AA Fantin VR. Detection of 2-hydroxyglutarate in IDH-mutated glioma patients by *in vivo* spectral-editing and 2D correlation magnetic resonance spectroscopy. Sci Transl Med (2012) 4(116):116ra4. doi: 10.1126/scitranslmed.3002693 PMC3720836 22238332

[33] Yu W Zhang L Wei Q Shao A. O6-Methylguanine-DNA methyltransferase (MGMT): Challenges and new opportunities in glioma chemotherapy. Front Oncol (2020) 9. doi: 10.3389/fonc.2019.01547 PMC6979006 32010632

[34] Szylberg M Sokal P Śledzińska P Bebyn M Krajewski S Szylberg L. MGMT promoter methylation as a prognostic factor in primary glioblastoma: A single-institution observational study. Biomedicines (2022) 10(8):2030. doi: 10.3390/biomedicines10082030 36009577 PMC9405779

[35] Sasaki T Kinoshita M Fujita K Fukai J Hayashi N Uematsu Y. Radiomics and MGMT promoter methylation for prognostication of newly diagnosed glioblastoma. Sci Rep (2019) 9(14435). doi: 10.1038/s41598-019-50849-y PMC6783410 31594994

[36] Chen S Xu Y Ye M Li Y Sun Y Liang J. Predicting MGMT promoter methylation in diffuse gliomas using deep learning with radiomics. J Clin Med (2022) 11(12):3445. doi: 10.3390/jcm11123445 35743511 PMC9224690

[37] Young RJ Gupta A Shah AD Graber JJ Zhang Z Shi W. Potential utility of conventional MRI signs in diagnosing pseudo-progression in glioblastoma. Neurology (2011) 76(22):1918–24. doi: 10.1212/WNL.0b013e31821d74e7 PMC3115805 21624991

[38] Abbasi AW Westerlaan HE Holtman GA Aden KM Jan van Laar P van der Hoorn A. Incidence of tumour progression and pseudoprogression in high-grade gliomas: a systematic review and meta-analysis. Clin Neuroradiol (2018) 28:401–11. doi: 10.1007/s00062-017-0584-x PMC6105173 28466127

[39] Thust SC van den Bent MJ Smits M. Pseudoprogression of brain tumors. JMRI (2018) 48(3):571–89. doi: 10.1002/jmri.26171 PMC6175399 29734497

[40] Taylor C Ekert JO Sefcikova V Fersht N Samandouras G. Discriminators of pseudoprogression and true progression in high-grade gliomas: A systematic review and meta-analysis. Sci Rep (2022) 12(1):13258. doi: 10.1038/s41598-022-16726-x 35918373 PMC9345984

[41] Bani-Sadr A Eker OF Berner LP Ameli R Hermier M Barritault M. Conventional MRI radiomics in patients with suspected early- or pseudo-progression. Neuro-Oncol Advances. (2019) 1(1). doi: 10.1093/noajnl/vdz019 PMC7212855 32642655

[42] Sun YZ Yan LF Han Y Nan HY Xiao G Tian Q. Differentiation of pseudoprogression from true progressionin glioblastoma patients after standard treatment: A machine learning strategy combinedwith radiomics features from T_1_-weighted contrast-enhanced imaging. BMC Med Imag (2021) 21(1):17. doi: 10.1186/s12880-020-00545-5 PMC7860032 33535988

[43] Elshafeey N Kotrotsou A Hassan A Elshafei N Hassan I Ahmed S. Multicenter study demonstrates radiomic features derived from magnetic resonance perfusion images identify pseudoprogression in glioblastoma. Nat Commun (2019) 10(3170). doi: 10.1038/s41467-019-11007-0 PMC6639324 31320621

[44] Saha A Yu X Sahoo D Mazurowski MA. Effects of MRI scanner parameters on breast cancer radiomics. Expert Syst Applicat (2017) 87:384–91. doi: 10.1016/j.eswa.2017.06.029 PMC6176866 30319179

[45] Lee J Steinmann A Ding Y Lee H Owens C Wang J. Radiomics feature robustness as measured using an MRI phantom. Sci Rep (2021) 11(1):3973. doi: 10.1038/s41598-021-83593-3 33597610 PMC7889870

[46] Saltybaeva N Tanadini-Lang S Vuong D Burgermeister S Mayinger M. Robustness of radiomic features in magnetic resonance imaging for patients with glioblastoma: Multi-center study. Phys Imaging Radiat Oncol (2022) 22:131–6. doi: 10.1016/j.phro.2022.05.006 PMC9130546 35633866

[47] Gidwani M Chang K Patel JB Hoebel KV Ahmed SR Singh P. Inconsistent partitioning and unproductive feature associations yield idealized radiomic models. Radiology (2022) 20:220715. doi: 10.1148/radiol.220715 PMC10068883 36537895

[48] Lohmann P Bousabarah K Hoevels M. Treuer H.Radiomics in radiation oncology–basics, methods, and limitations. Strahlentherapie und Onkolo (2020) 196:848–55. doi: 10.1007/s00066-020-01663-3 PMC7498498 32647917

[49] Wagner MW Namdar K Biswas A Monah S Khalvati F Ertl-Wagner BB. Radiomics, machine learning, and artificial intelligence-what the neuroradiologist needs to know. Neuroradiology (2021) 63(12):1957–67. doi: 10.1007/s00234-021-02813-9 PMC8449698 34537858

[50] Zwanenburg A Vallières M Abdalah MA Aerts HJWL Andrearczyk V Apte A. The image biomarker standardization initiative: Standardized quantitative radiomics for high-throughput image-based phenotyping. Radiology (2020) 295(2):328–38. doi: 10.1148/radiol.2020191145 PMC7193906 32154773

[51] Liu X Li Y Qian Z Sun Z Xu K Wang K. A radiomic signature as a non-invasive predictor of progression-free survival in patients with lower-grade gliomas. NeuroImage Clin (2018) 20:1070–7. doi: 10.1016/j.nicl.2018.10.014 PMC6202688 30366279

[52] Franco P Würtemberger U Dacca K Hübschle I Beck J Schnell O. SPectroscOpic prediction of bRain tumours (SPORT): study protocol of a prospective imaging trial. BMC Med Imag (2020) 20(1):123. doi: 10.1186/s12880-020-00522-y PMC7685595 33228567

[53] Lohmann P Franceschi E Vollmuth P Dhermain F Weller M. Radiomics in neuro-oncological clinical trials. Lancet (2022) 4(11):E841–9. doi: 10.1016/S2589-7500(22)00144-3 36182633

[54] Tomaszewski MR Gillies RJ. The biological meaning of radiomics features. Radiology (2021) 298(3):505–16. doi: 10.1148/radiol.2021202553 PMC7924519 33399513

